# Supplementary material for: BAP1 dysregulation impairs trophoblast differentiation and contributes to placental dysfunction in preeclampsia
Source: Cell Death Dis. 2026 Mar 26;17(1):410. doi: 10.1038/s41419-026-08650-z (PMC13144379; doi:10.1038/s41419-026-08650-z)
Supplement: Supplementary file 1 — Supplementary Figures and Legends [file 41419_2026_8650_MOESM1_ESM.docx]

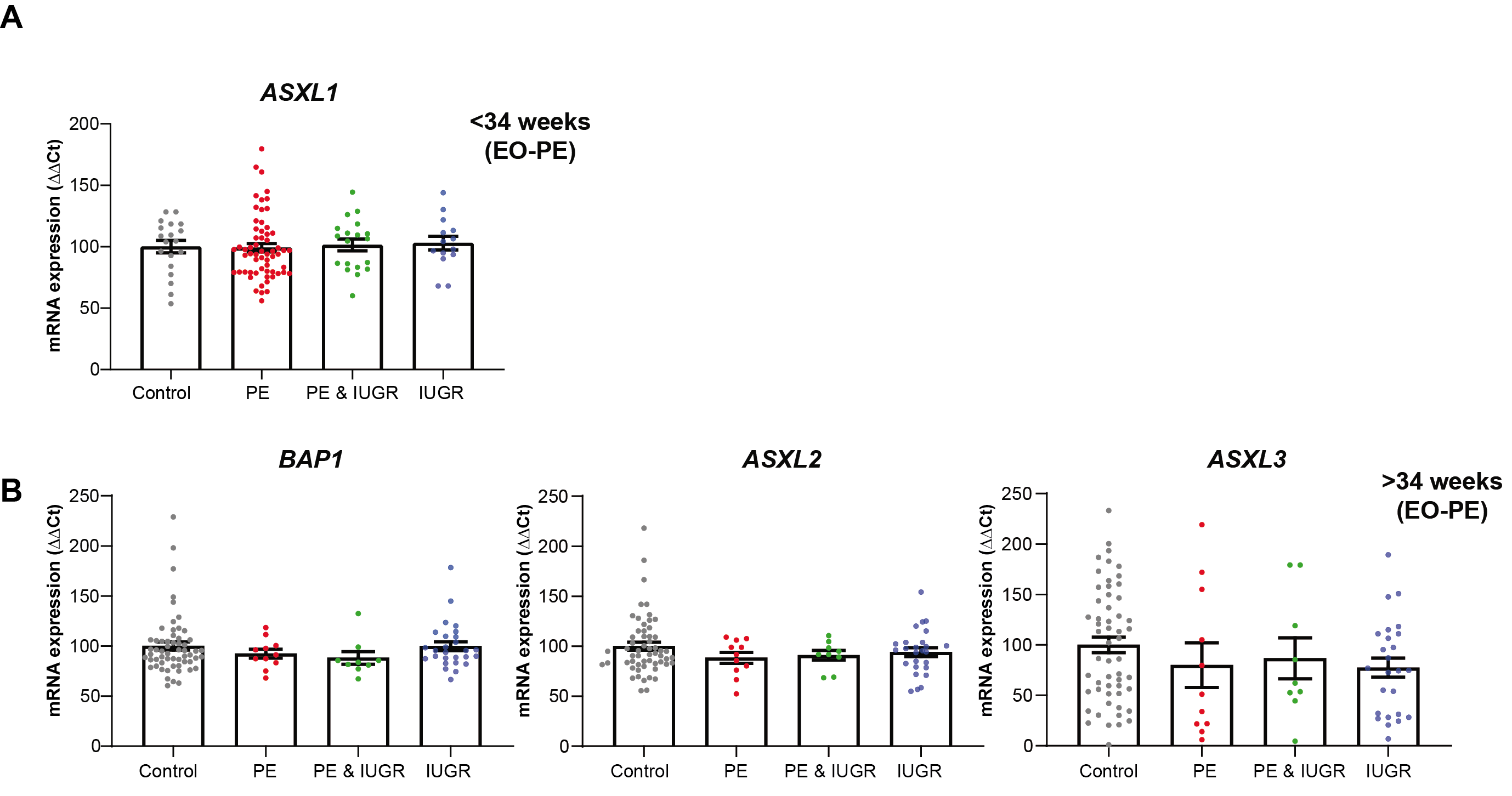


**Supplementary Figure 1:** **BAP1 PR-DUB complex levels in placental tissue from pregnancies with complications. (A)** Relative mRNA expression of ***ASXL1*** in central villous placental biopsies collected before 34 weeks of gestation from gestational age–matched control pregnancies (Control PT; preterm, n=30), preeclampsia (PE, n=56), PE with intrauterine growth restriction (PE&IUGR, n=19), or IUGR alone (n=12). **(B)** Relative mRNA expression of *BAP1*, ***ASXL2* and *ASXL3*** in central villous placental biopsies collected after 34 weeks of gestation from gestational age–matched control pregnancies (Control; n=57), preeclampsia (PE, n=11), PE with intrauterine growth restriction (PE&IUGR, n=8), or IUGR alone (n=27).


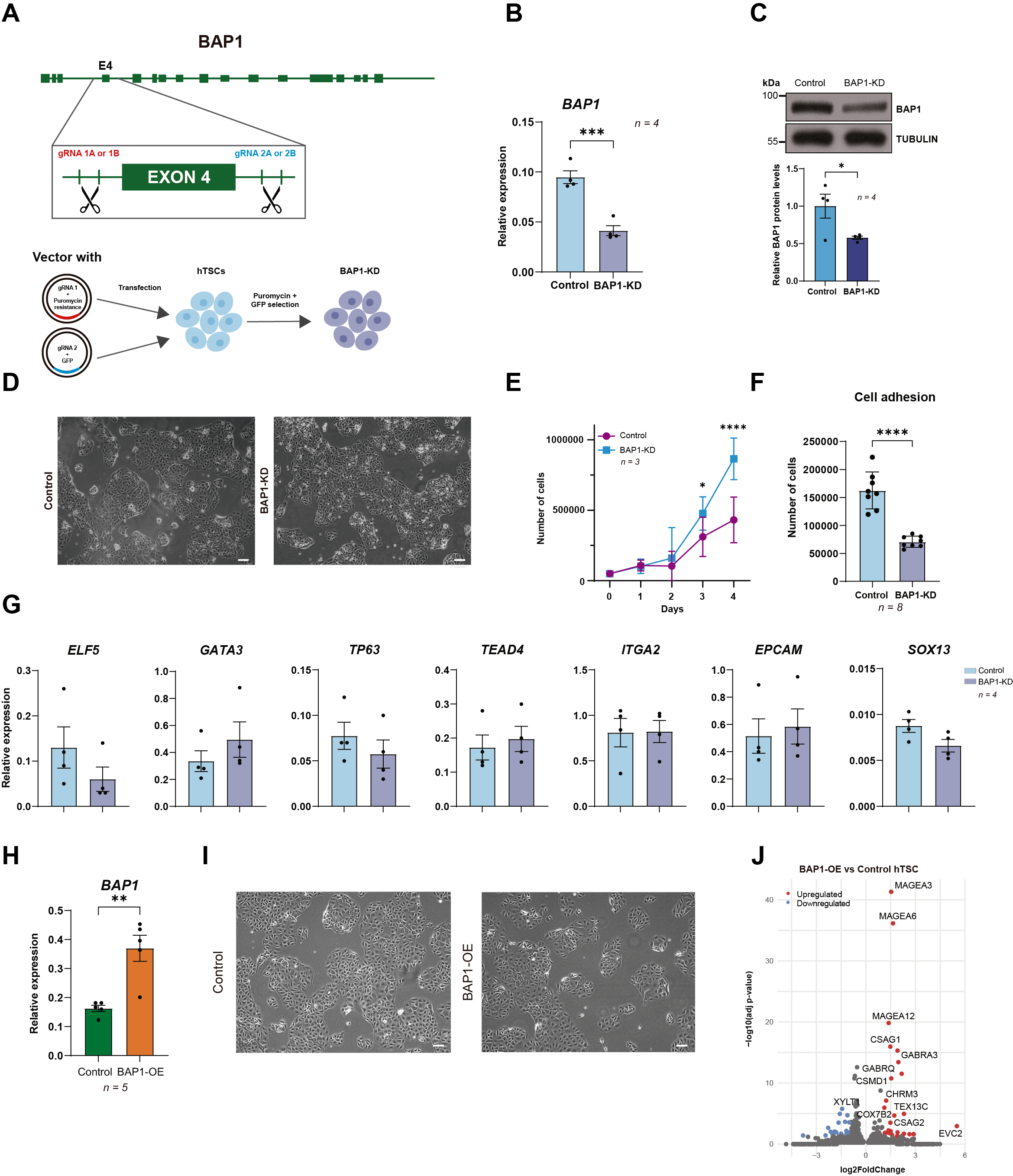


**Supplementary Figure 2: BAP1 knockdown and BAP1 overexpression alter trophoblast cell biology.** (**A**) CRISPR-Cas9 targeting strategy for generating BAP1-knockdown (KD) hTSCs, showing guide RNA (gRNA) positioning in introns surrounding exon 4. (**B**) RT-qPCR analysis of *BAP1* (exon4) levels in BAP1-KD and control hTSCs (mean ± SEM, *n=*4 independent experiments; ***p<0.001; Student’s two-tailed t-test. (**C**) Western blot analysis of BAP1 and loading control (TUBULIN) in BAP1-KD versus Control hTSCs. **Quantification of relative BAP1 protein levels**, normalized to the corresponding loading control and expressed relative to control cells, is shown at the bottom (mean ± SEM, n = 4 independent experiments; p < 0.05; Student’s two-tailed t-test). (**D**) Phase-contrast images demonstrating conserved morphology in BAP1-KD *vs* Control hTCSs. Scale bars = 150µm. (**E**) Proliferation rates of BAP1-KD and control hTSCs over 4 days. BAP1 depletion significantly increased cell numbers at days 3 and 4 (mean ± SEM, *n=*3 independent experiments; *p<0.05, ****p<0.0001; two-way ANOVA followed by Holm-Sidak’s post hoc test). (**F**) Adhesion assay quantifying attached BAP1-KD and control hTSCs (mean ± SEM, *n*=8 independent experiments; ****p<0.0001, Student’s two-tailed t-test). (**G**) RT-qPCR analysis of and stem cell (*ELF5,* *GATA3, TP63, TEAD4, SOX13*) and epithelial (*EPCAM, ITGA2*) markers in BAP1-KD versus control hTSCs. Data represent mean ± SEM (*n=*4 independent experiments; Student’s two-tailed t-test). (**H**) RT-qPCR analysis of *BAP1* expression in BAP1-OE versus Control hTSCs (mean ± SEM, *n*=5 independent experiments; **p<0.01; Student’s two-tailed t-test­­­). (**I**) Phase-contrast images confirming conserved morphology in BAP1-OE hTSCs. Scale bar = 150 µm. (**J**) Volcano plot of differentially expressed genes (DEGs) in BAP1-OE hTSCs versus controls (red: upregulated genes; blue: downregulated genes; adjusted *p*< 0.05, |log2FC| > 1). hTSC, human trophoblast stem cell; OE, overexpression.


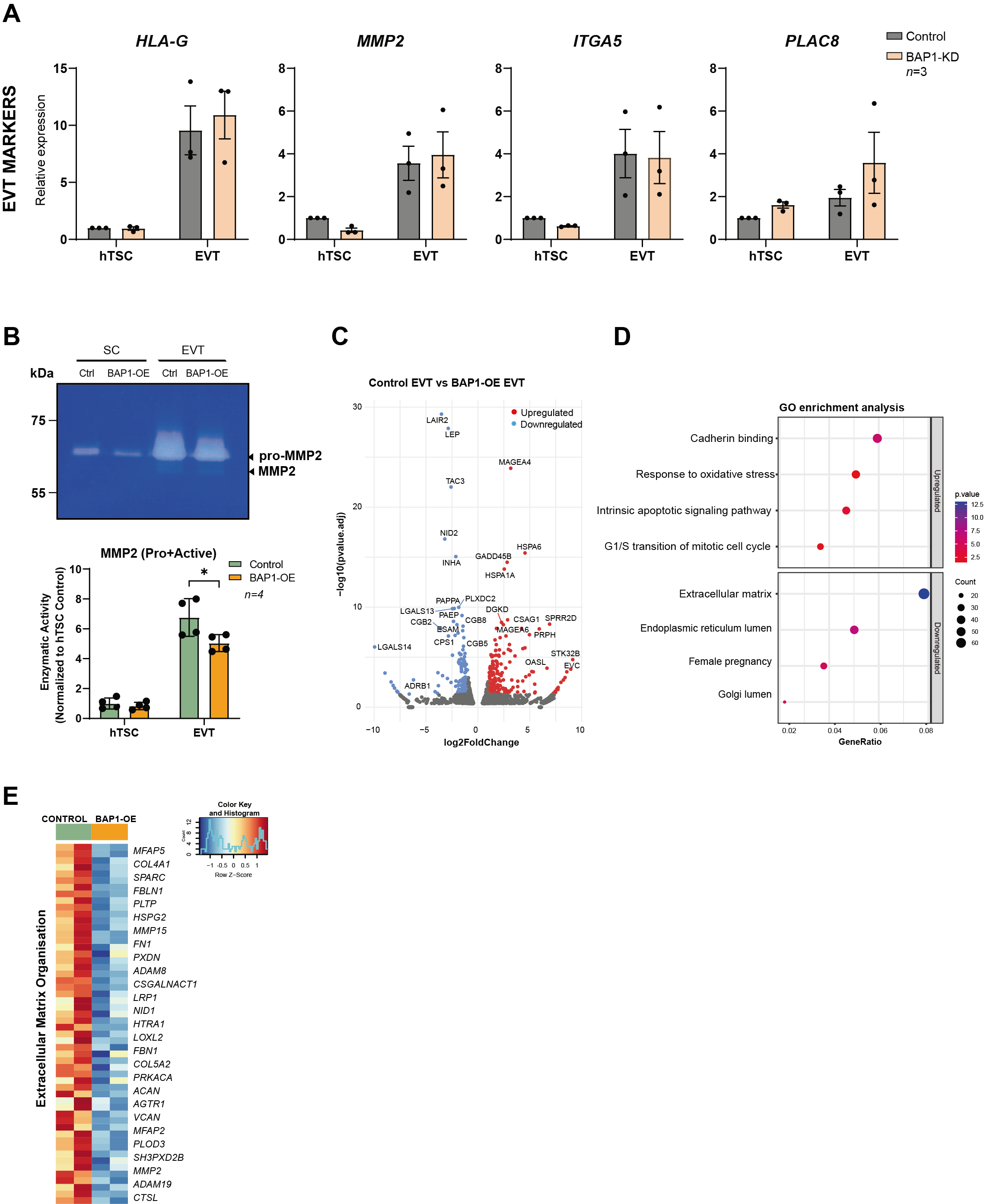


**Supplementary Figure 3: BAP1 overexpression impairs extravillous trophoblast differentiation.** (**A**) RT-qPCR analysis of extravillous trophoblast (EVT) markers (*HLA-G*, *MMP2, ITGA5*, and *PLAC8*) in BAP1-KD human trophoblast stem cells (hTSC) and EVTs compared to controls. Data are mean ± SEM, *n=*3 independent experiments, two-way ANOVA with Tukey’s multiple comparisons test. (**B**) Gelatin zymography demonstrating reduced MMP2 proteolytic activity in BAP1-OE hTSCs and EVTs compared to control cells. On the bottom, quantification of MMP2 activity (normalized to hTSC controls; mean ± SEM, *n*=4 independent experiments; *p<0.05, two-way ANOVA with Tukey’s multiple comparisons test). (**C**) Volcano plot of differentially expressed genes (DEGs) in BAP1-OE EVTs versus control EVTs (red: upregulated genes; blue: downregulated genes; adjusted *p*< 0.05, |log2FC| > 1). (**D**) Functional enrichment analysis of DEGs, highlighting pathways associated with upregulated and downregulated genes. Circle size represents gene count; color indicates statistical significance (-log10(p-value)). (**E**) Heatmap of FPKM-normalized expression for extracellular matrix (ECM)-related genes, showing dysregulated expression patterns in BAP1-OE EVTs (row-scaled Z-scores). OE, overexpressed.


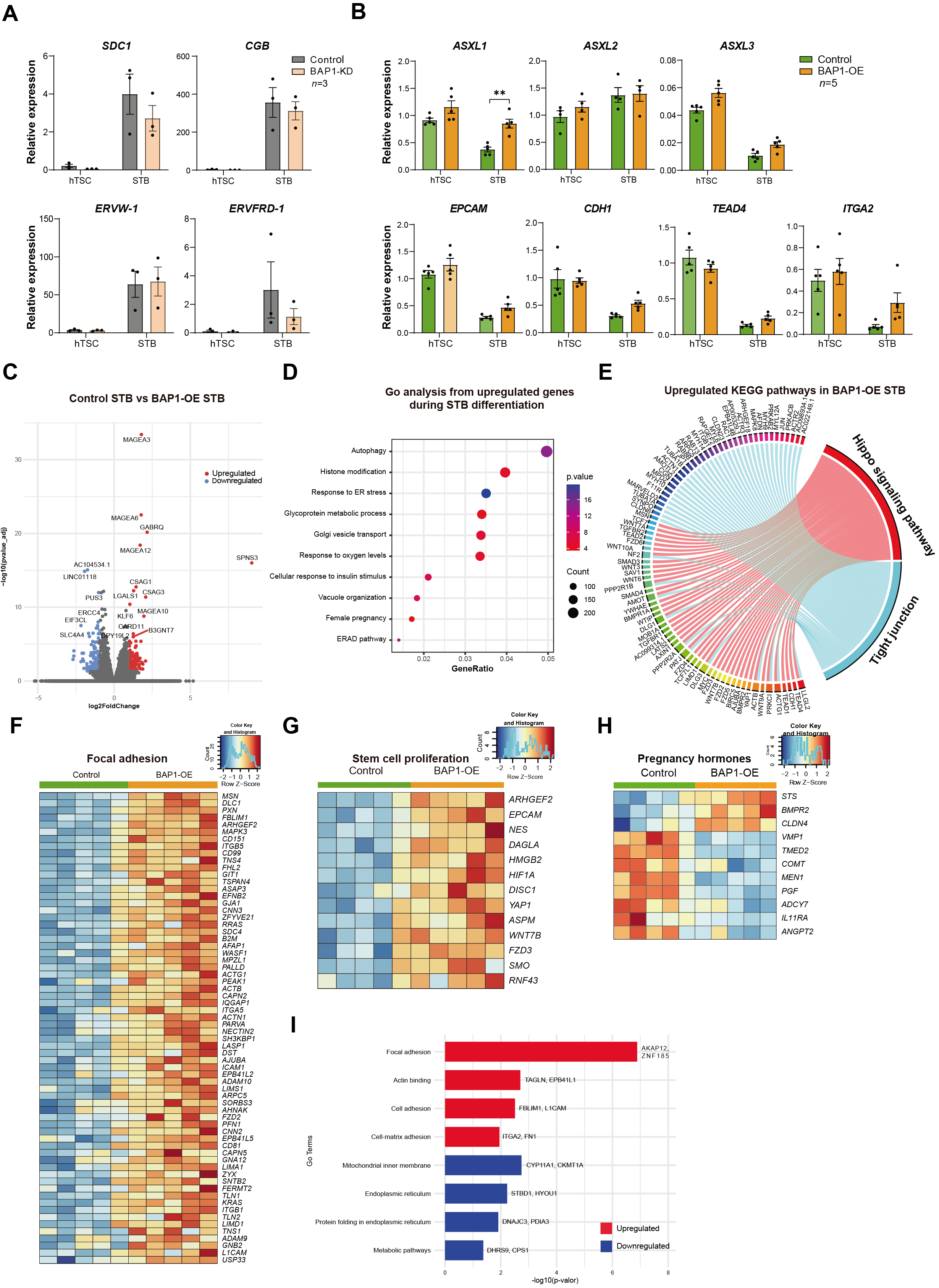


**Supplementary Figure 4: BAP1 overexpression disrupts syncytiotrophoblast differentiation.** (**A**) RT-qPCR analysis of syncytiotrophoblast (STB) markers (*SDC1*, *CGB*, *ERVW-1*, *ERVFRD-1*) in BAP1-KD hTSCs and STBs compared to controls. Data are mean ± SEM*, n=*3 independent experiments, two-way ANOVA with Tukey’s multiple comparisons test. (**B**) RT-qPCR analysis of PR-DUB components (*ASXL1-3*), epithelial (*EPCAM*, *CDH1*) and stem cell (*TEAD4*, *ITGA2*) markers in BAP1-OE hTSC and STBs compared to controls (mean ± SEM of *n=*5 independent experiments; ***p*<0.005, two-way ANOVA with Tukey’s multiple comparisons test). (**C**) Volcano plot of significantly upregulated (red) and downregulated (blue) genes (|log2FC>1|, adjusted *p*<0.05) in BAP1-OE compared control STBs. (**D**) Gene Ontology (GO) enrichment analysis (bubble plot) of upregulated pathways upon normal STB differentiation. Circle size represents gene count; color indicates statistical significance (-log10(p-value)). (**E**) Chord diagram illustrating key upregulated pathways in BAP1-OE STBs compared to Control STBs. (**F-H**) Heatmaps of FPKM values for genes related to (**F**) focal adhesion, (**G**) stem cell proliferation, and (**H**) pregnancy hormones in BAP1-OE and control samples. Rows are z-score normalized. (**I**) Functional enrichment analysis of DEPs, highlighting pathways associated with upregulated (red) and downregulated (blue) proteins. hTSC, human trophoblast stem cell; OE, overexpression.


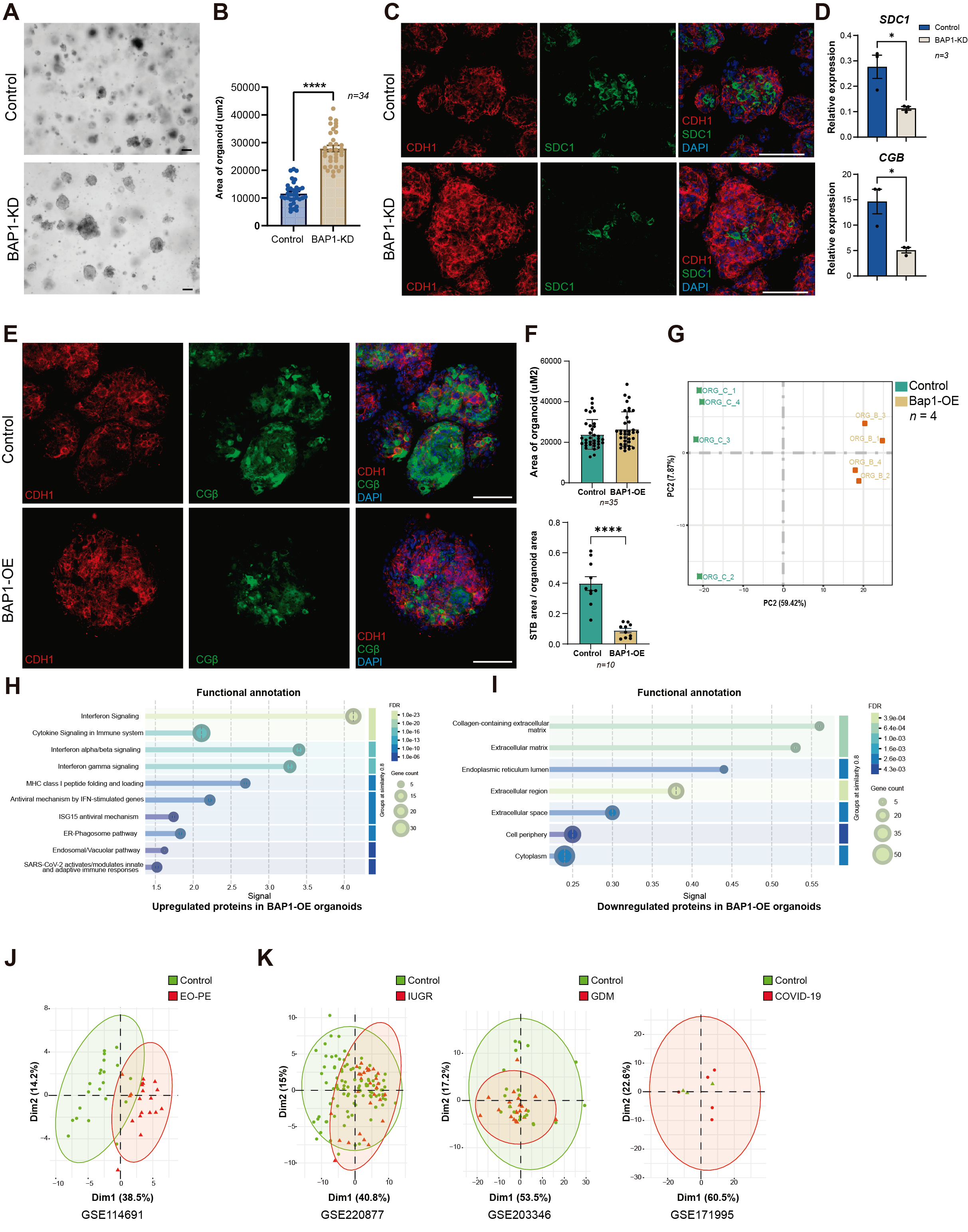


**Supplementary Figure 5: BAP1 overexpression disrupts trophoblast organoid formation and recapitulates early onset-preeclampsia features** (**A**) Brightfield images demonstrating increased organoid size in BAP1-KD compared to controls. Scale bars, 100 µm. (**B**) Quantification of total organoid area, confirming morphological expansion in BAP1-KD conditions (mean ± SEM, *n=*34 organoids/group; p****<0.0001; Student’s two-tailed t-test) (**C**) Immunofluorescence analysis of epithelial (CDH1) and syncytiotrophoblast (SDC1) markers in BAP1-KD organoids compared to controls, with DAPI nuclear counterstain. Scale bars, 100 µm. (**D**) RT-qPCR profiling of syncytiotrophoblast markers (*SDC1, CGB*) in BAP1-KD versus control organoids (mean ± SEM, *n=*3 independent experiments; *p<0.05; Student’s two-tailed t-test). (**E**) Immunofluorescence analysis of BAP1-OE and control organoids for CDH1 and CGβ. Nuclear counterstaining was with DAPI. Scale bars, 150 μm. (**F**) Quantification of organoid cross-sectional area (upper graph) shows no significant differences between groups (mean ± SEM; n = 35; Student’s two-tailed t-test). The lower graph shows the ratio of syncytiotrophoblast (STB) area to total organoid area, revealing a significant reduction in STB formation in BAP1-OE organoids (mean ± SEM; n = 10; **** p < 0.0001; Student’s two-tailed t-test). (**G**) Principal component analysis (PCA) of RNA-seq data showing distinct clustering of BAP1-overexpressing and control organoids, reflecting global transcriptional differences. (**H-I**) STRING functional annotation analysis of (**H**) upregulated and (**I**) downregulated pathways in BAP1-OE organoids compared to control organoids. Circle size represents gene count; color indicates False Discovery Rate (FDR). (**J-K**) Principal component analysis (PCA) plots showing separation between control (green) and disease (red) placental samples from **EO-PE (GSE114691)** (**J**), and **IUGR (GSE220877)**, **GDM (GSE203346)**, and **COVID-19 (GSE171995)** datasets (**K**), based on the BAP1-OE molecular signature. Axes represent principal components 1 and 2, explaining the indicated percentage of total variance. OE, overexpression.
