# Supplementary material for: BAP1 dysregulation impairs trophoblast differentiation and contributes to placental dysfunction in preeclampsia
Source: Cell Death Dis. 2026 Mar 26;17(1):410. doi: 10.1038/s41419-026-08650-z (PMC13144379; doi:10.1038/s41419-026-08650-z)
Supplement: Supplementary file 2 — Original data [file 41419_2026_8650_MOESM2_ESM.pdf]

Figure 1C

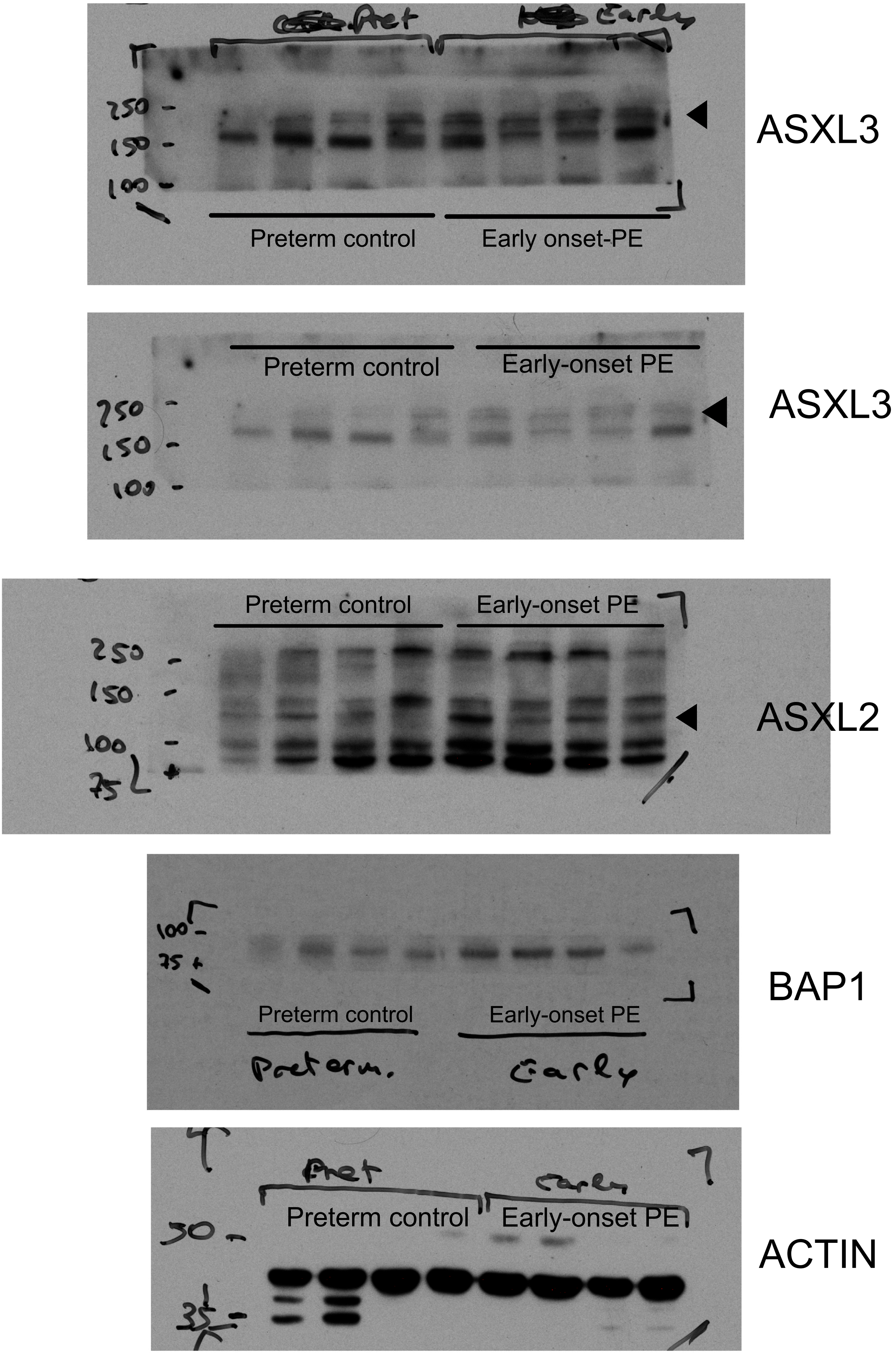

Preterm control
Early onset-PE
ASXL3
Preterm control
Early-onset PE
ASXL3
Preterm control
Early-onset PE
ASXL2
Preterm control
Early-onset PE
BAP1
Preterm control
Early-onset PE
ACTIN

Figure 1D

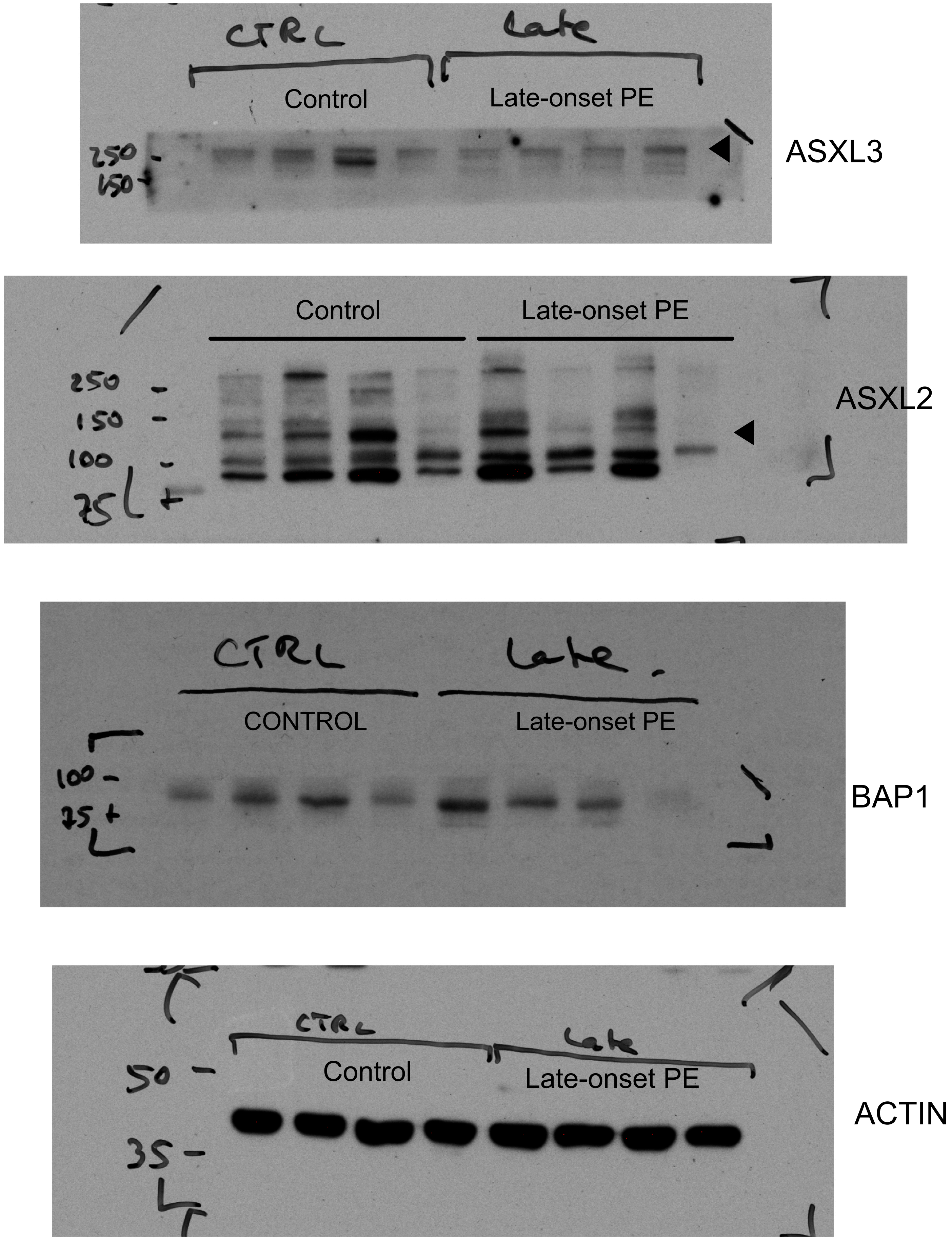
CTRL
Late
Control
Late-onset PE
250
150
ASXL3
Control
Late-onset PE
250
150
100
75
ASXL2
CTRL
Late
CONTROL
Late-onset PE
100
75
BAP1
CTRL
Late
Control
Late-onset PE
50
35
ACTIN

Figure 2B

N=1

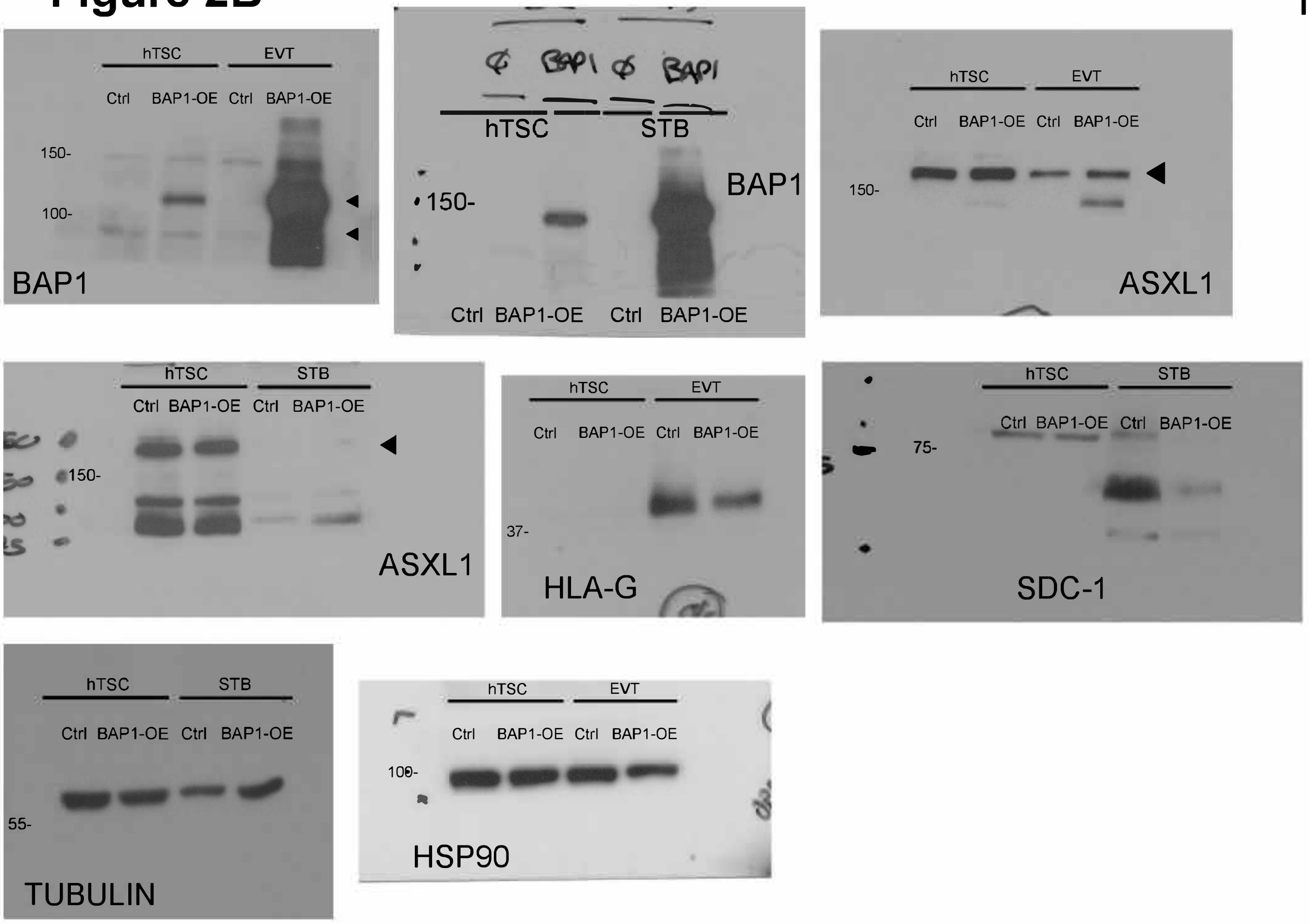

N=2

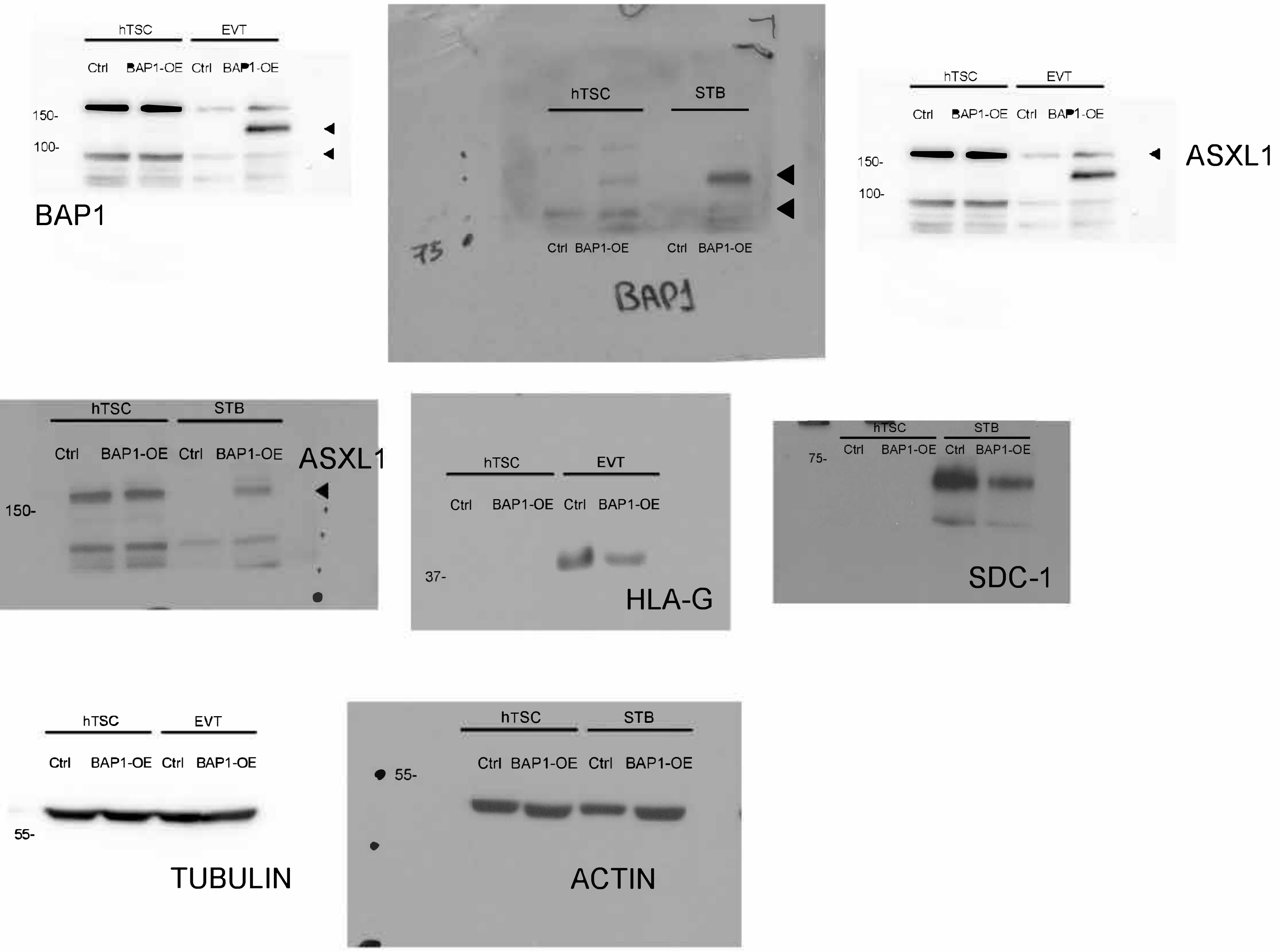

Figure 2B

N=3

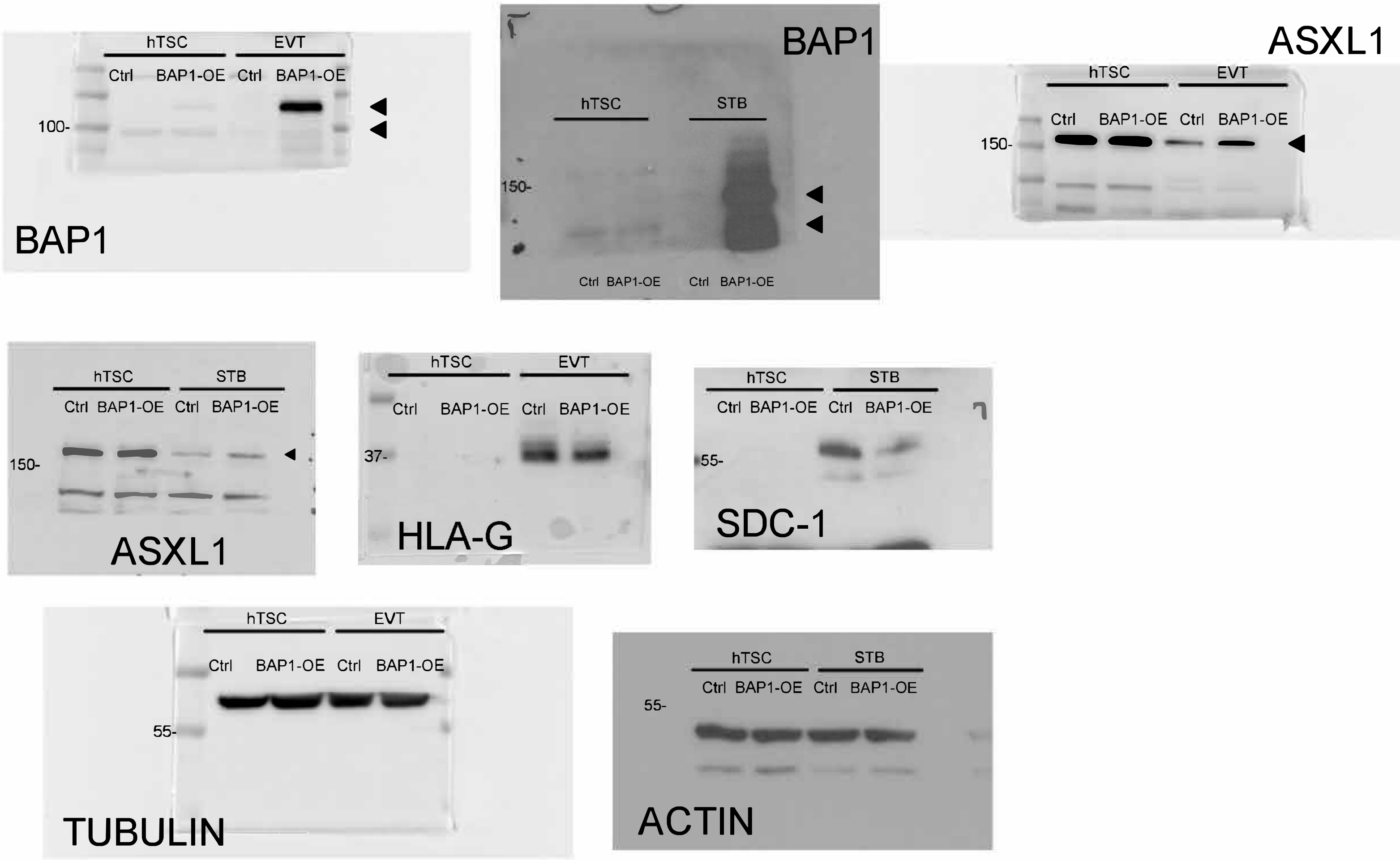

N=4

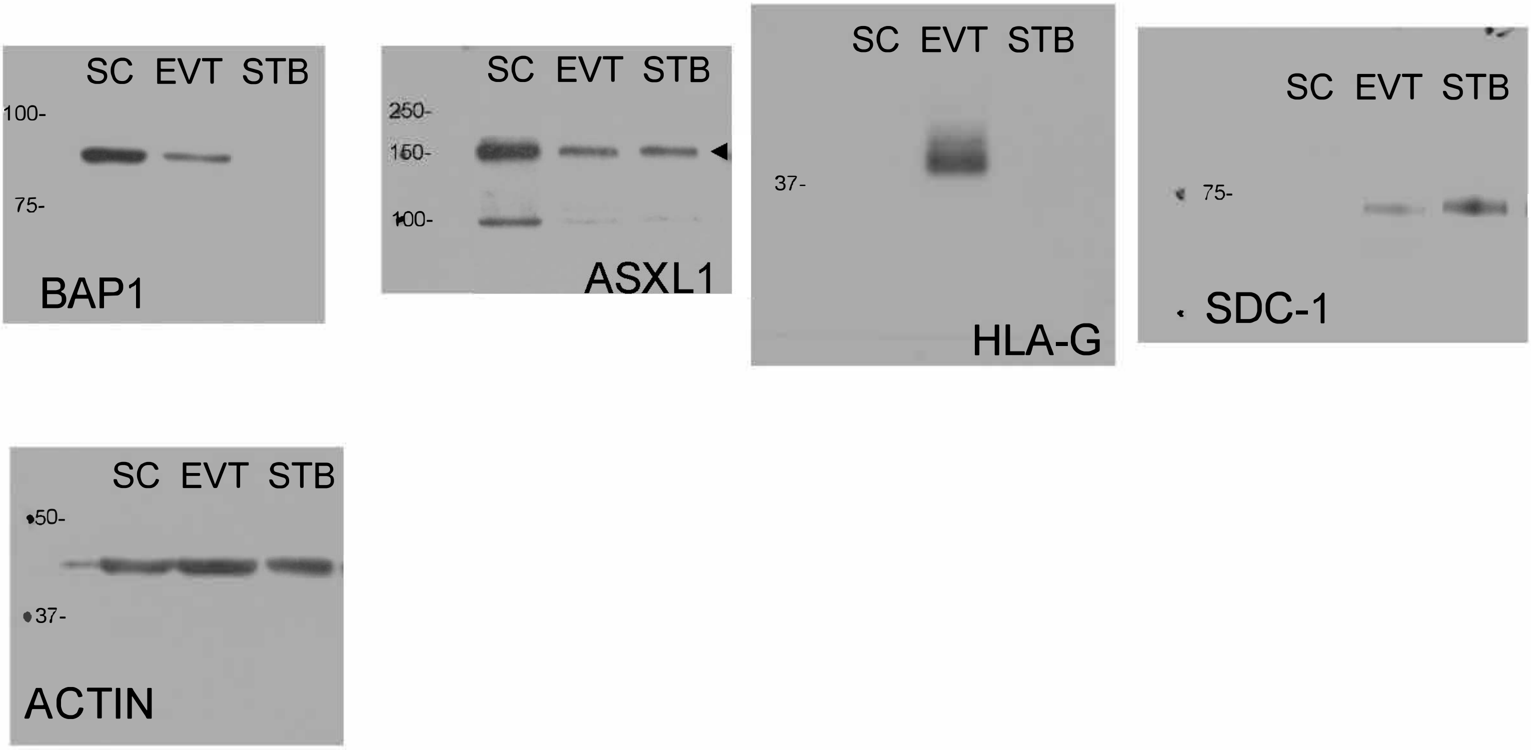

Figure 2C

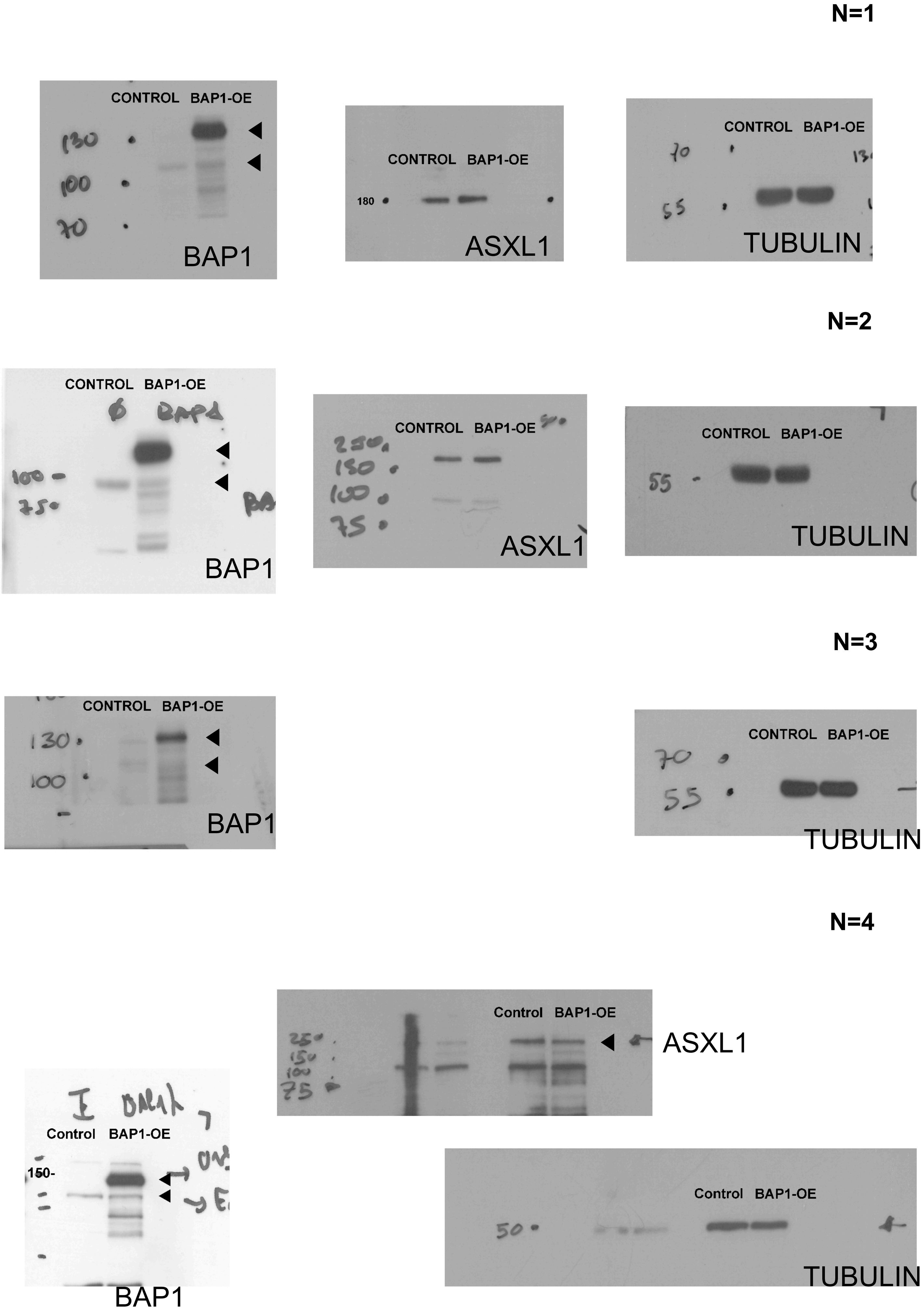
N=1
CONTROL BAP1-OE
BAP1
CONTROL BAP1-OE
180
ASXL1
CONTROL BAP1-OE
TUBULIN
N=2
CONTROL BAP1-OE
BAP1
CONTROL BAP1-OE
ASXL1
CONTROL BAP1-OE
TUBULIN
N=3
CONTROL BAP1-OE
BAP1
CONTROL BAP1-OE
TUBULIN
N=4
Control BAP1-OE
ASXL1
Control BAP1-OE
150-
BAP1
Control BAP1-OE
TUBULIN

Figure 3B

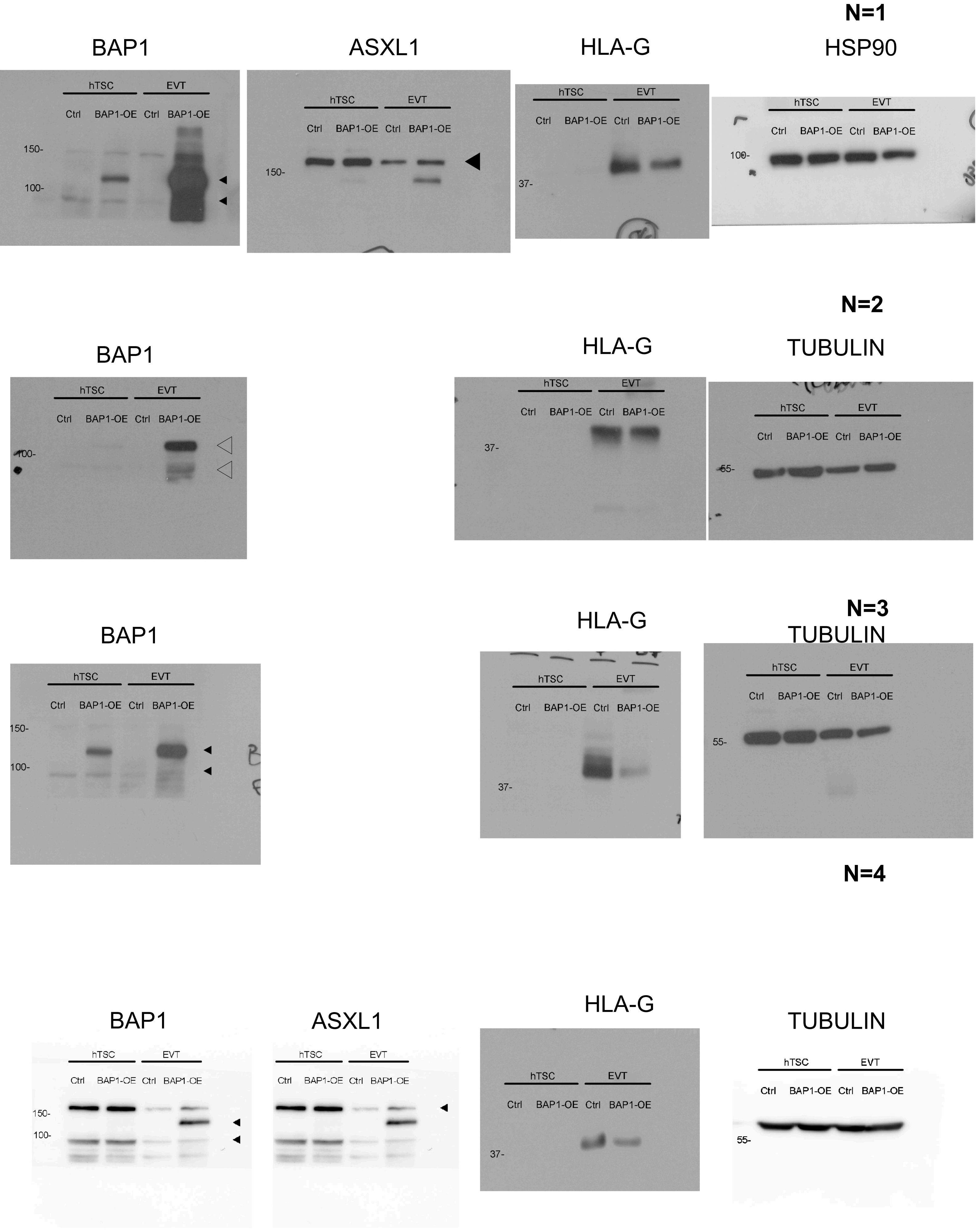
N=1
BAP1
ASXL1
HLA-G
HSP90
hTSC
EVT
Ctrl
BAP1-OE
150-
100-
37-
N=2
BAP1
HLA-G
TUBULIN
55-
N=3
BAP1
HLA-G
TUBULIN
N=4
BAP1
ASXL1
HLA-G
TUBULIN

Figure 3B

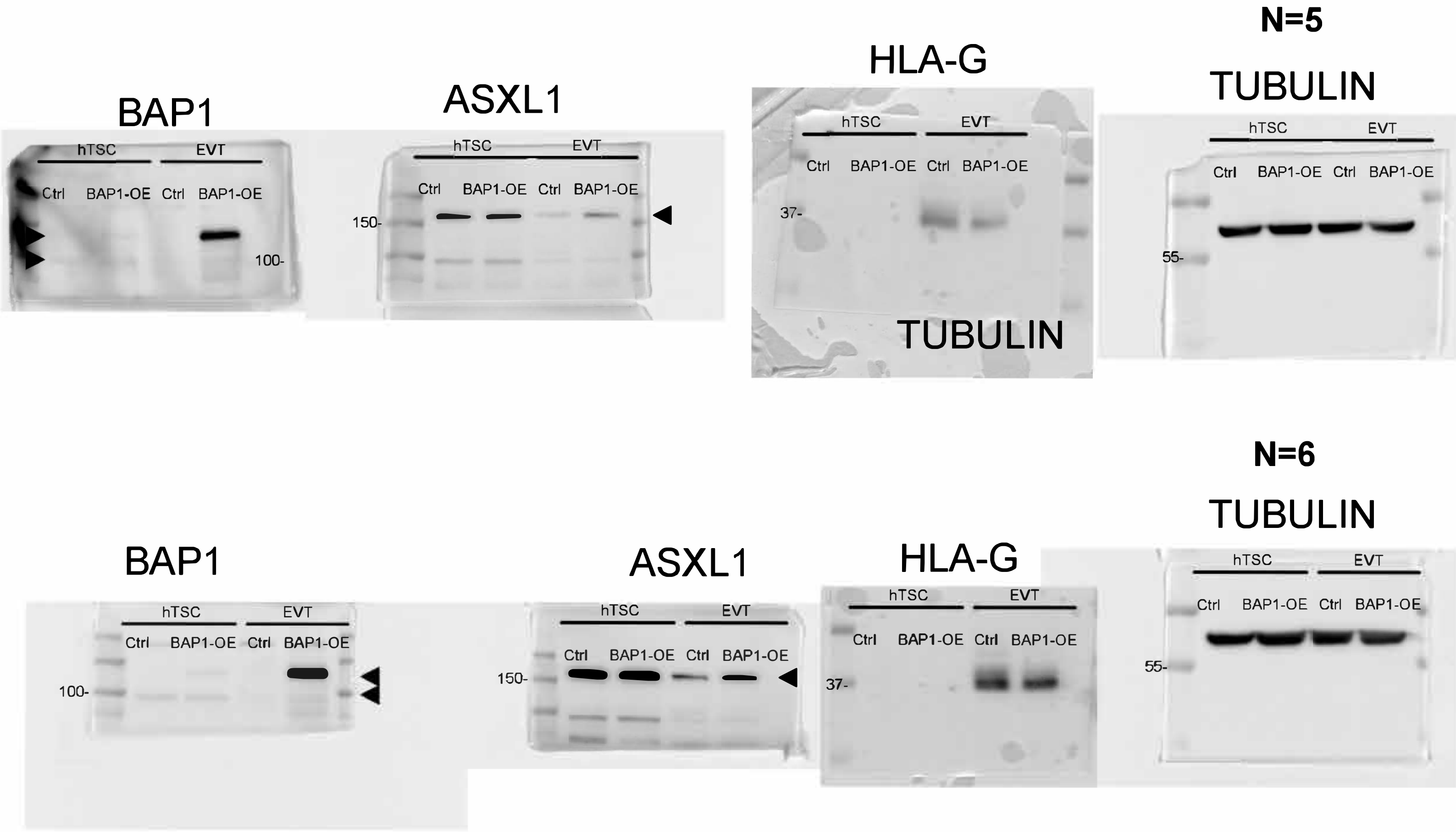
N=5
BAP1
hTSC
EVT
Ctrl
BAP1-OE
Ctrl
BAP1-OE
100-
ASXL1
hTSC
EVT
Ctrl
BAP1-OE
Ctrl
BAP1-OE
150-
HLA-G
hTSC
EVT
Ctrl
BAP1-OE
Ctrl
BAP1-OE
37-
TUBULIN
TUBULIN
hTSC
EVT
Ctrl
BAP1-OE
Ctrl
BAP1-OE
55-
N=6
BAP1
hTSC
EVT
Ctrl
BAP1-OE
Ctrl
BAP1-OE
100-
ASXL1
hTSC
EVT
Ctrl
BAP1-OE
Ctrl
BAP1-OE
150-
HLA-G
hTSC
EVT
Ctrl
BAP1-OE
Ctrl
BAP1-OE
37-
TUBULIN
hTSC
EVT
Ctrl
BAP1-OE
Ctrl
BAP1-OE
55-

Figure 4B

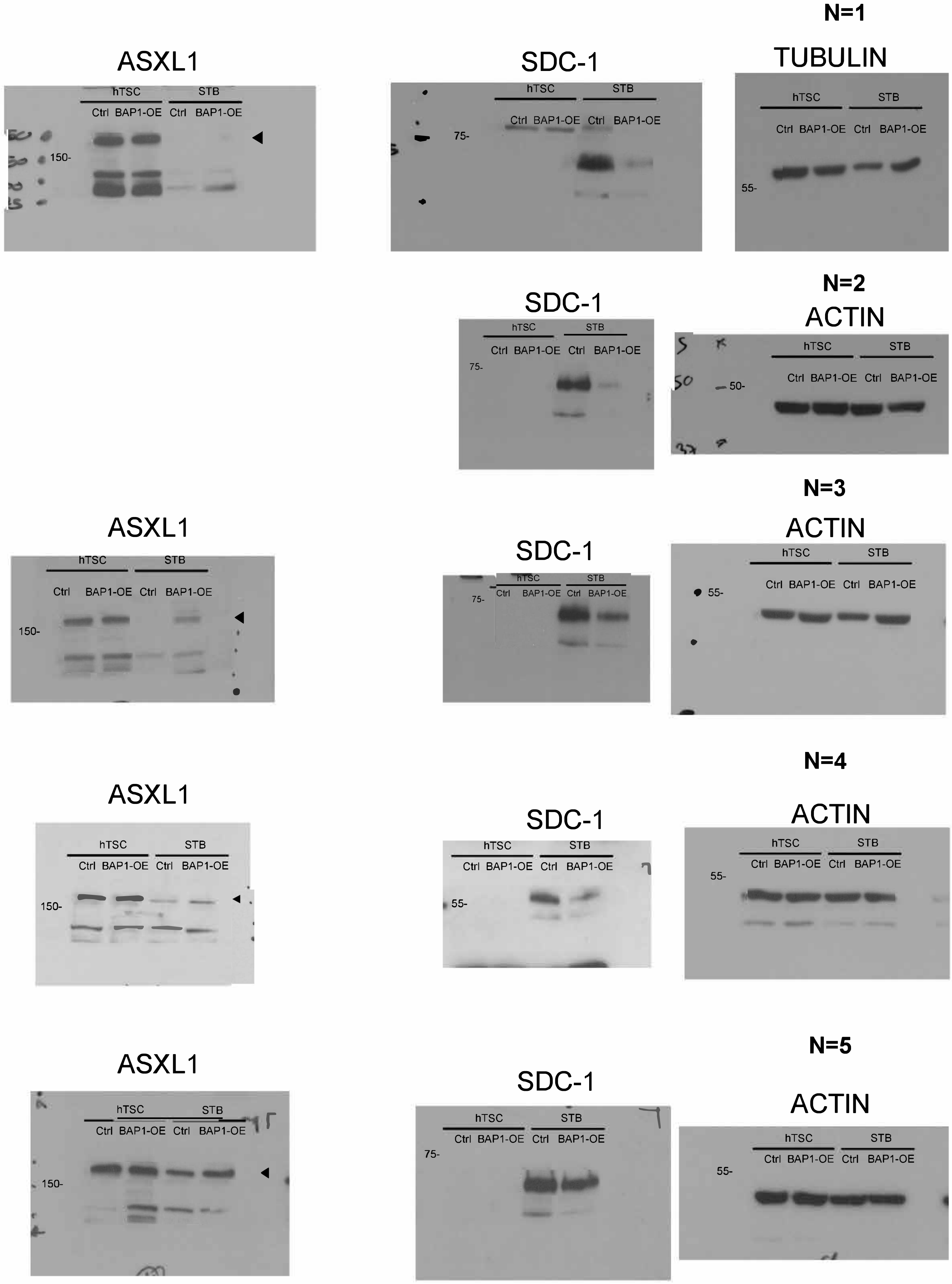
N=1
ASXL1
hTSC
STB
Ctrl BAP1-OE Ctrl BAP1-OE
150-
SDC-1
75-
TUBULIN
55-
N=2
SDC-1
ACTIN
50-
N=3
ASXL1
SDC-1
ACTIN
55-
N=4
ASXL1
SDC-1
ACTIN
N=5
ASXL1
SDC-1
ACTIN

Figure 5D

N=1

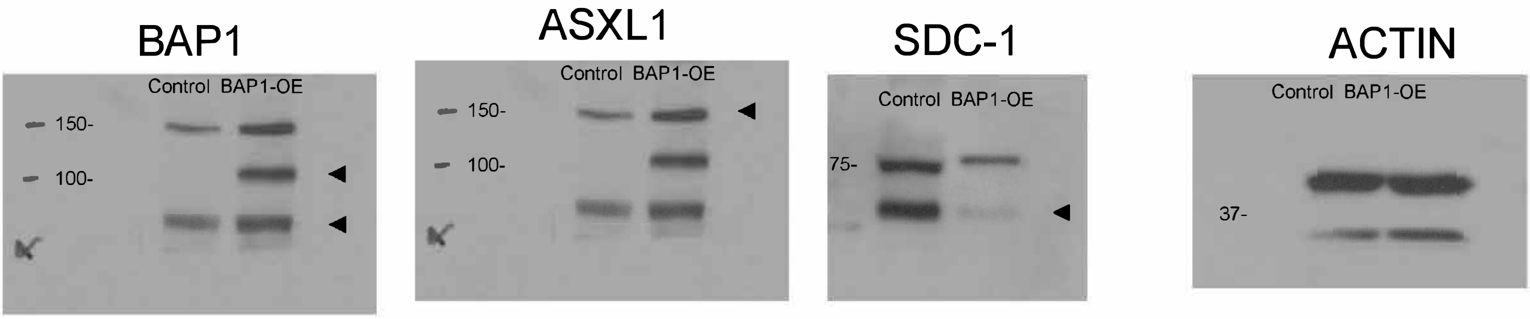

N=2-5

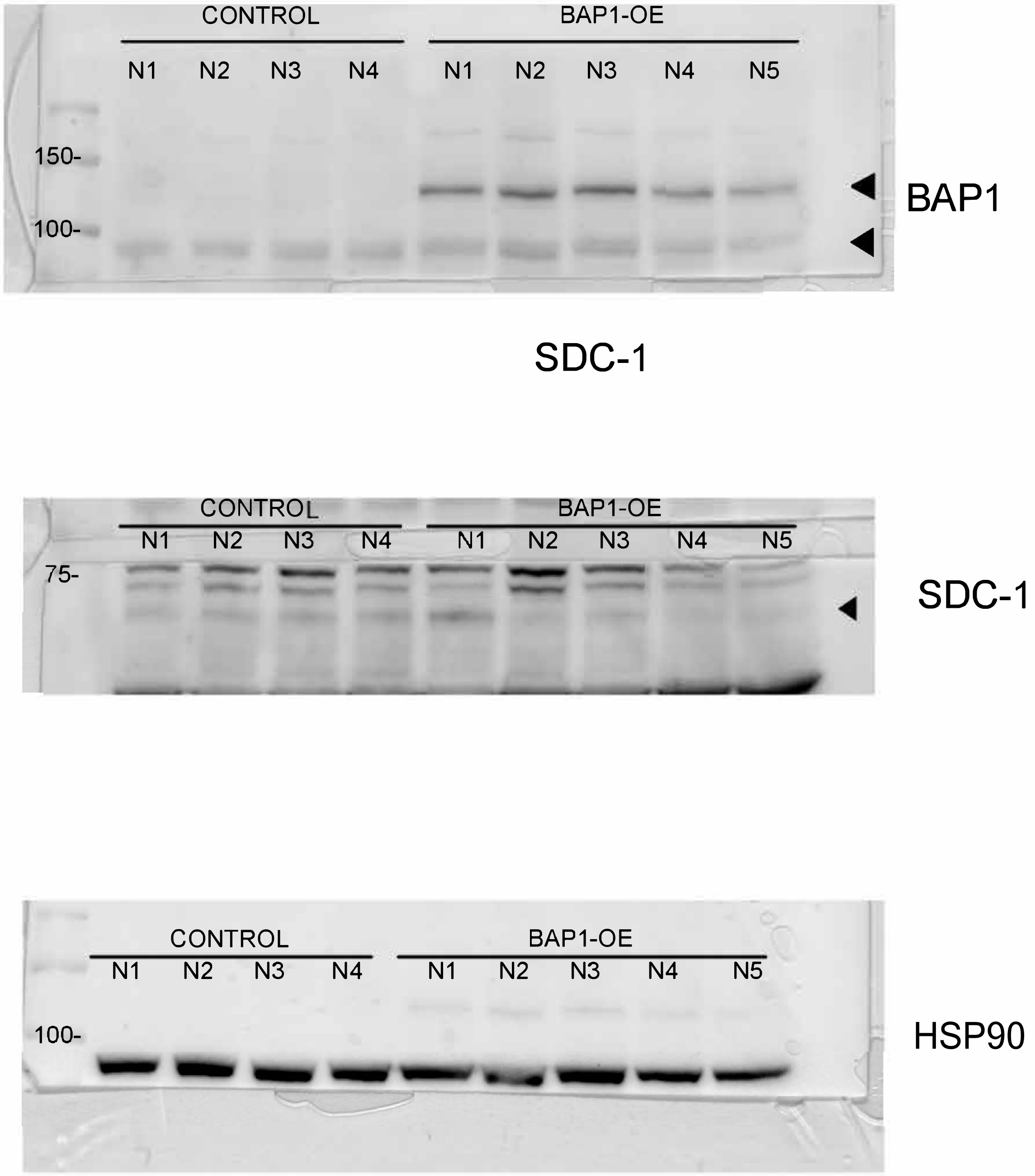

Figure S2C

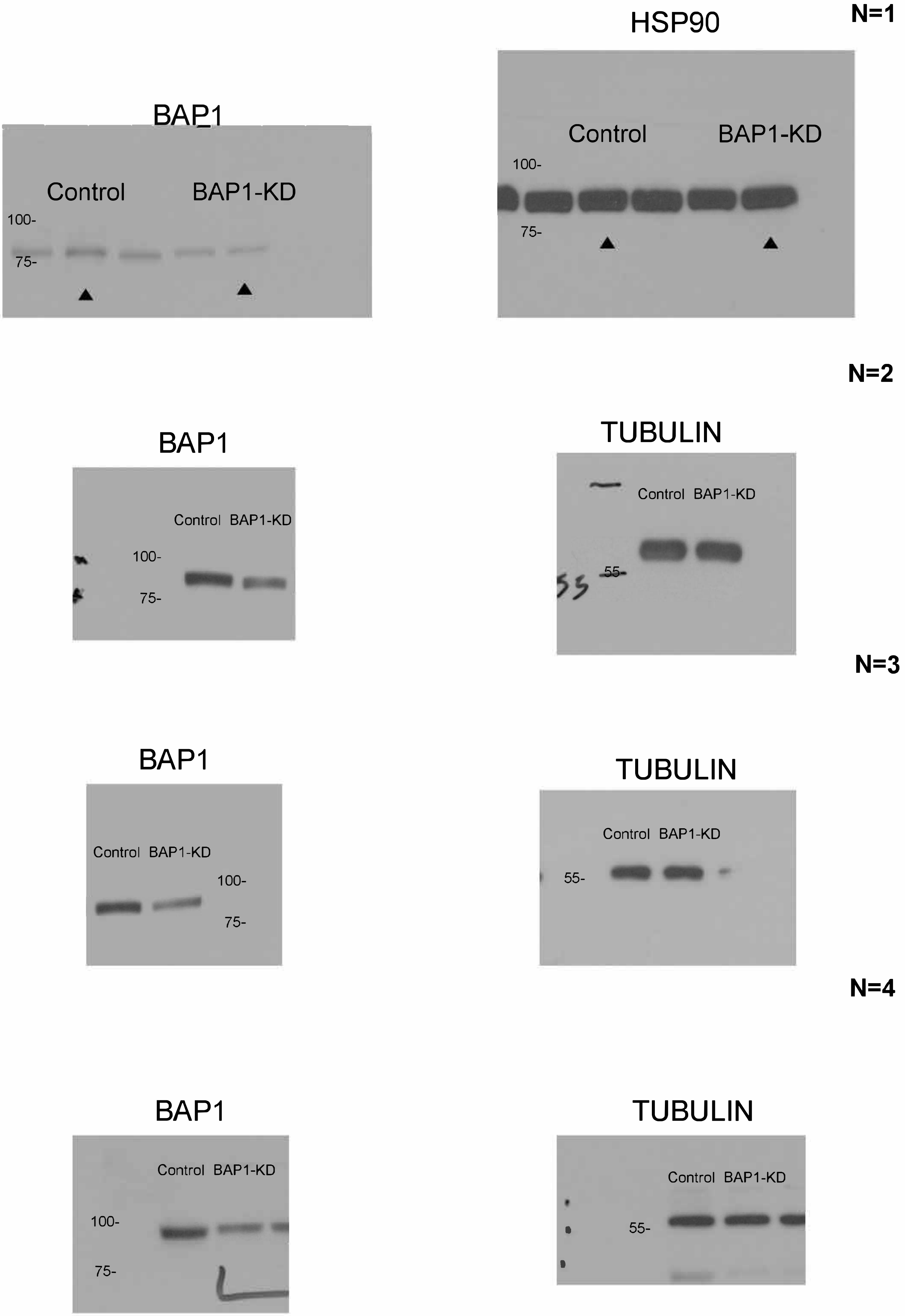
N=1
BAP1
Control
BAP1-KD
100-
75-
HSP90
Control
BAP1-KD
100-
75-
N=2
BAP1
Control BAP1-KD
100-
75-
TUBULIN
Control BAP1-KD
55-
N=3
BAP1
Control BAP1-KD
100-
75-
TUBULIN
Control BAP1-KD
55-
N=4
BAP1
Control BAP1-KD
100-
75-
TUBULIN
Control BAP1-KD
55-
